# Comparison of Four Ultrasonography-Based Risk Stratification Systems in Thyroid Nodules with Nondiagnostic/Unsatisfactory Cytology: A Real-World Study

**DOI:** 10.3390/cancers13081948

**Published:** 2021-04-18

**Authors:** You-Bin Lee, Young-Lyun Oh, Jung-Hee Shin, Sun-Wook Kim, Jae-Hoon Chung, Yong-Ki Min, Soo-Yeon Hahn, Tae-Hyuk Kim

**Affiliations:** 1 Samsung Medical Center, Department of Medicine, Division of Endocrinology and Metabolism, Sungkyunkwan University School of Medicine, 81 Irwon-ro, Gangnam-gu, Seoul 06351, Korea; yb.snowyday1224@gmail.com (Y.-B.L.); sunwooksmc.kim@samsung.com (S.-W.K.); jaeh.chung@samsung.com (J.-H.C.); ykm.min@samsung.com (Y.-K.M.); 2 Samsung Medical Center, Department of Pathology, Sungkyunkwan University School of Medicine, 81 Irwon-ro, Gangnam-gu, Seoul 06351, Korea; yl.oh@samsung.com; 3 Samsung Medical Center, Department of Radiology, Sungkyunkwan University School of Medicine, 81 Irwon-ro, Gangnam-gu, Seoul 06351, Korea; helena35.shin@samsung.com

**Keywords:** thyroid, fine-needle aspiration, nondiagnostic, unsatisfactory, thyroid imaging, reporting and data system (TIRADS)

## Abstract

**Simple Summary:**

Although ultrasound-based risk stratification systems (RSSs) including Thyroid Imaging, Reporting and Data Systems (TIRADSs) may play an important role in triaging nodules with nondiagnostic/unsatisfactory cytology, no previous studies have compared ultrasound-based RSSs for these nodules. In this retrospective, longitudinal, real-world study in Korea including 1143 thyroid aspirations with nondiagnostic/unsatisfactory results from 1125 patients, further diagnostic evaluations, including repeat fine-needle aspiration, were conducted more commonly as the categories of ultrasound-based RSSs increased. The American Thyroid Association (ATA) guidelines, Korean (K)-TIRADS, and American College of Radiology (ACR) TIRADS were more competent in predicting malignancy from nondiagnostic/unsatisfactory nodules. The EU-TIRADS, although it was also helpful, demonstrated less effective diagnostic performance in predicting malignancy for nondiagnostic/unsatisfactory nodules in Korea, where iodine intake is more than adequate. These findings have implications for developing and verifying universal guidelines for the ultrasound-based stratification of thyroid nodules and applying these guidelines to nondiagnostic/unsatisfactory nodules.

**Abstract:**

We compared American Thyroid Association (ATA) guidelines, Korean (K)-Thyroid Imaging, Reporting and Data Systems (TIRADS), EU-TIRADS, and American College of Radiology (ACR) TIRADS in diagnosing malignancy for thyroid nodules with nondiagnostic/unsatisfactory cytology. Among 1143 nondiagnostic/unsatisfactory aspirations from April 2011 to March 2016, malignancy was detected in 39 of 89 excised nodules. The minimum malignancy rate was 7.82% in EU-TIRADS 5 and 1.87–3.00% in EU-TIRADS 3–4. In the other systems, the minimum malignancy rate was 14.29–16.19% in category 5 and ≤3% in the remaining categories. Although the EU-TIRADS category ≥ 5 exhibited the highest positive likelihood ratio (LR) of only 2.214, category ≥ 5 in the other systems yielded the highest positive LR of >5. Receiver operating characteristic (ROC) curves of all systems to predict malignancy were located statistically above the diagonal nondiscrimination line (P for ROC curve: EU-TIRADS, 0.0022; all others, 0.0001). The areas under the ROC curve (AUCs) were not significantly different among the four systems. The ATA guidelines, K-TIRADS, and ACR TIRADS may be useful to guide management for nondiagnostic/unsatisfactory nodules. The EU-TIRADS, although also useful, exhibited inferior performance in predicting malignancy for nondiagnostic/unsatisfactory nodules in Korea, an iodine-sufficient area.

## 1. Introduction

For individuals with thyroid nodules, ultrasound (US) is a primary diagnostic modality to evaluate the risk of malignancy (ROM) and to inform decisions regarding the application of fine-needle aspiration (FNA) [1]. For the effective management of thyroid nodules, systems to guide US practitioners in recommending FNA based on US features have been proposed by professional societies or investigators [1,2,3,4,5]. For some of these US-based risk stratification systems (RSSs), the terminology of the Thyroid Imaging, Reporting and Data System (TIRADS) has been used [3]. These US-based RSSs include the nodule sonographic pattern system proposed by the 2015 revised American Thyroid Association (ATA) guidelines [4], the Korean TIRADS (K-TIRADS) by the Korean Thyroid Association (KTA)/Korean Society of Thyroid Radiology (KSThR) in 2016 [1,2], the European (EU)-TIRADS by the European Thyroid Association (ETA) in 2017 [5], and the American College of Radiology (ACR) TIRADS in 2017 [3]. Regarding these US-based RSSs that have been widely utilized in different areas of the world, there have been studies that compared their diagnostic performance [6,7,8,9,10,11,12]. Although a meta-analysis reported better performance for the ACR TIRADS than the ATA nodule sonographic pattern system or K-TIRADS in selecting nodules for FNA, comparisons across the commonly used systems were limited by the limited data availability [8].

FNA represents the standard tool to triage patients with thyroid nodules for excision or clinical observation [13,14]. Thyroid cytopathology reports have been standardized according to the Bethesda system for reporting thyroid cytopathology (TBSRTC), consisting of six diagnostic tiers with certain ROM ranges and management guidelines [15,16]. Of these six categories, the nondiagnostic/unsatisfactory (ND/UNS) is the main limitation [17,18]. Although it is suggested as ideal to limit the ND/UNS results to <10% of all cytopathologic specimens according to TBSRTC [15], a much higher percentage of ND/UNS cases (up to 23.6%) was reported in a meta-analysis [14]. Although the experience of aspirators and cytopathologists and onsite adequacy assessments may affect the inadequacy rate, the characteristics of the nodule itself are also important determinators [18]. In patients with technically challenging nodules, it can be difficult to obtain an adequate sample even with repeat aspirations [14,18,19], linked to the relatively large proportion of ND/UNS specimens in large academic/reference centers. For proper management, the ROM of nodules with inadequate specimens should be maximally predicted with given information, including US findings. Therefore, US-based RSSs may play a particularly important role in triaging ND/UNS nodules [17,20,21]. It is necessary to assess the US-based RSSs of different guidelines and to compare their diagnostic performance for ND/UNS nodules to establish effective evidence-based recommendations for nodules with inadequate specimens and to facilitate communication between radiologists, endocrinologists, surgeons, and pathologists regarding the management of these diagnostically challenging nodules. However, to the best of our knowledge, no studies have compared various US-based RSSs for ND/UNS nodules. Therefore, we determined and compared the utility of US-based RSSs (the ATA nodule sonographic pattern system, K-TIRADS, EU-TIRADS, and ACR TIRADS) in diagnosing malignancy for ND/UNS nodules.

## 2. Materials and Methods

### 2.1. Case Selection

**Figure 1:**
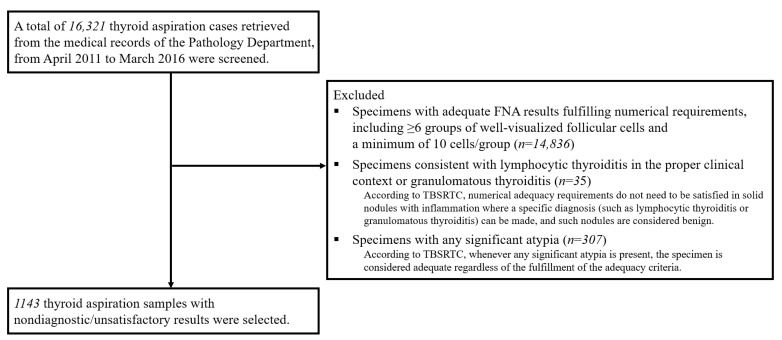
Steps for case selection. TBSRTC = the Bethesda system for reporting thyroid cytopathology [15,16].

This study was conducted at Samsung Medical Center, a hospital-based tertiary referral center in Korea. This study was approved by the Institutional Review Board (IRB) of Samsung Medical Center (SMC 2020-12-069). The IRB waived the requirement for informed consent because all data were deidentified. TBSRTC was adopted by our pathology department in April 2011 [13,18]. From a total of 16,321 thyroid aspiration cases retrieved from the medical records of the Pathology Department, from April 2011 to March 2016, only ND/UNS specimens were selected after excluding those with adequate FNA results according to the steps described in Figure 1. Finally, the remaining 1143 thyroid aspiration samples from 1125 patients were enrolled.

### 2.2. Specimen Preparation and US Examinations

Methods of US examinations and specimen preparation have been described in our previous studies [13,18].

### 2.3. Review of US Images According to the US-Based RSSs

We retrospectively reviewed all US images obtained on the same day as aspirations and analyzed the sonographic features of the nodules while blinded to the final diagnosis. These features were interpreted following the 2015 revised ATA [4], 2016 KTA/KSThR [2], 2017 ETA [5], and 2017 ACR [3] guidelines. All nodules were categorized according to the ATA nodule sonographic pattern system [4], K-TIRADS [2], EU-TIRADS [5], and ACR TIRADS [3]. For effective communication, five groups of sonographic patterns categorized by the ATA guidelines were assigned to five category levels (category 5 for high suspicion of malignancy; category 4 for intermediate suspicion of malignancy; category 3 for low suspicion of malignancy; category 2 for very low suspicion of malignancy; and category 1 for benign). For the K-TIRADS, EU-TIRADS, and ACR TIRADS, their own TIRADS categories were used.

### 2.4. Histological Follow-Up and Determination of Final Diagnoses

Participants had follow-ups until March 2020, and whether surgical resection was performed was confirmed. The final diagnosis of malignancy was based on the histopathological findings from surgical resection. We calculated the malignancy rate in two ways (maximum and minimum) [13,18,22]. The maximum malignancy rate, estimated only for resected nodules, was derived by dividing the number of surgically confirmed malignant nodules by the number of nodules selected for surgical resection. The minimum malignancy rate was determined by dividing the number of histopathologically confirmed malignant nodules by the total number of nodules aspirated, regardless of whether surgical resection was applied. Noninvasive follicular thyroid neoplasms with papillary-like nuclear features (NIFTPs) were not regarded as malignancies [23].

For the final diagnosis of benign nodules, the satisfaction of ≥one of the followings was required [24]: (1) benign histopathological findings after surgical resection; (2) benign pathological results from follow-up core needle biopsy (CNB); (3) benign cytological results from follow-up FNA procedures repeated at least twice; and (4) benign findings of FNA with a stable size on follow-up. Based on this definition, the benign rate was also calculated in two ways (maximum and minimum). The maximum benign rate was the percentage of benign cases calculated from the total number of nodules with cytological or histopathological follow-up data obtained by repeat FNA, CNB, and/or surgical resection. The minimum benign rate was the percentage of cases calculated from the total number of aspirated nodules.

### 2.5. Statistical Analyses

The baseline characteristics of the patients and nodules were analyzed with respect to all cases and cases treated with surgical resection. Continuous variables are shown as the median and interquartile range, while categorical variables are expressed as the frequency and percentage. The maximum and minimum malignancy/benign rates were calculated according to the categories of US-based RSSs. After including surgically-resected cases only, the positive and negative likelihood ratios (LRs) for category cutoffs of US-based RSSs in diagnosing malignancy were calculated according to the established method [25,26]. Since it is essential to use the gold standard in calculating the LR to evaluate the performance of diagnostic tests, the gold standard diagnosis based on surgical pathology was applied, including surgically-resected nodules only. The greater the LR is from 1.0, the greater the increase in the probability of malignancy [25]. LRs from 2 to 5 correspond to small increases in the posttest probability of malignancy, LRs from 5 to 10 indicate moderate increases, and LRs > 10 suggest that a positive test is adequate for ruling in a diagnosis of malignancy [25]. For LRs < 1.0, the smaller the LR is, the greater its effect is on the decrease in the probability of malignancy [25]. For surgically-resected nodules, the area under the curve (AUC) was obtained from receiver operating characteristic (ROC) curve analyses to assess the performances of US-based RSSs in predicting malignancy. In calculating the LRs and analyzing the ROC curves for the ATA nodule sonographic pattern system, three unclassifiable cases (isoechoic/hyperechoic nodules with ≥one of the following features: irregular margins, microcalcification, taller-than-wide shape, rim calcification with small extrusive soft tissue component, and evidence of extrathyroidal extension) were excluded from analyses. Pairwise comparisons of AUCs of US-based RSSs were conducted using the DeLong test [27]. Pairwise comparisons including ATA nodule sonographic pattern system were conducted after excluding three unclassifiable cases for the ATA guideline. MedCalc statistical software version 19.5.3 (MedCalc Software, Ostend, Belgium) was used for statistical analyses. The level of significance was set at *p* < 0.05 for two-tailed tests.

## 3. Results

### 3.1. Characteristics of the Study Population and Nodules

During the five-year period, 1143 ND/UNS aspiration samples from 1125 patients were included (Table 1). The median age of the population was 54.0 years. Of the 1125 individuals, 843 (74.90%) were females. The median nodule size was 1.30 cm. Of 1143 thyroid nodules, 89 (7.79%) were surgically-resected.

### 3.2. Malignancy Rates According to Categories of US-Based RSSs

The malignancy rates are presented according to the categories of US-based RSSs (Table 2). Of the 89 excised nodules, 39 were malignant on surgical pathology, yielding maximum and minimum malignancy rates of 43.82% and 3.41%, respectively. Among these 39 malignant nodules, 32 (82.1%) were papillary thyroid carcinomas (PTCs), 6 (15.4%) were follicular thyroid carcinomas (FTCs), and the rest one nodule (2.6%) was medullary thyroid carcinoma (MTC) (Table S1). With respect to all US-based RSSs, as the category advanced, the minimum malignancy rate tended to increase. Under the ATA nodule sonographic pattern system, K-TIRADS, and ACR TIRADS, category 5, compared to the other categories, was associated with a marked increase in the maximum and minimum malignancy rates. Category 5 of the ATA nodule sonographic pattern system, K-TIRADS, and ACR TIRADS showed minimum malignancy rates of 14.29–16.19%, while the other categories of these three US-based RSSs exhibited minimum malignancy rates of ≤3%. However, the minimum malignancy rate in category 5 of the EU-TIRADS was 7.82%, demonstrating a smaller difference from the values of categories 3–4 (1.87–3.00%).

### 3.3. Benign Rates According to Categories of US-Based RSSs

The benign rates are summarized according to the categories of US-based RSSs (Table 3). Among the 1143 thyroid nodules, 206 (18.02%) were followed-up cytologically or histopathologically through repeat FNA, CNB, and/or surgical resection. Of the 206 nodules with cytological or histopathological follow-up data, 115 were confirmed as benign at final diagnosis, demonstrating benign rates of 55.83% (maximum) and 10.06% (minimum). The proportion of cytological or histopathological follow-up was higher in more advanced categories than in less advanced categories. Under the ATA nodule sonographic pattern system, K-TIRADS, and ACR TIRADS, 32–35% of nodules were followed-up cytologically or histopathologically in category 5, whereas 19–20% were followed-up in category 4, and only 10–15% were followed-up in category 3. The proportion of cytological or histopathological follow-up was 25.51% in category 5, 18.67% in category 4, and 14.94% in category 3 of the EU-TIRADS. When we applied the ATA nodule sonographic pattern system, K-TIRADS, or ACR TIRADS, the maximum benign rate of category 5 was 30.56–33.33%, demonstrating less than half of the maximum benign rate in category 4 (67.03–68.18%). When analyzed in the EU-TIRADS, the maximum benign rate was 46.77% in category 5 and 66.07% in category 4.

### 3.4. LRs for US-Based RSSs in Diagnosing Thyroid Malignancy

Table 4 presents the positive and negative LRs of US-based RSSs in diagnosing malignancy. Applying the cutoff of category ≥ 5 yielded the highest positive LRs of ≥5 in the ATA nodule sonographic pattern system, K-TIRADS, and ACR TIRADS (5.368, 5.449, and 5.128, respectively). However, the EU-TIRADS showed the highest positive LR of only 2.214 when the cutoff of category ≥ 5 was applied.

### 3.5. ROC Curves of US-Based RSSs to Predict Thyroid Malignancy

**Figure 2:**
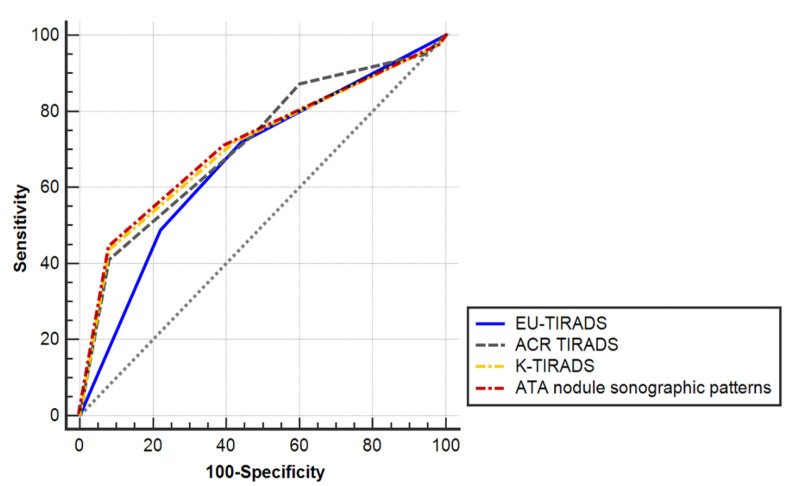
Receiver operating characteristic (ROC) curves of four ultrasonography-based risk stratification systems to predict thyroid malignancy in nodules with nondiagnostic or unsatisfactory cytopathological results.

The ROC curves of all US-based RSSs in predicting malignancy were located statistically above the diagonal nondiscrimination line (Figure 2 and Table 5, *p* for the ROC curve of the EU-TIRADS = 0.0022, all others *p* = 0.0001). The differences in the AUC of US-based RSSs are summarized in Table 6. There were no significant differences in the AUC among the four US-based RSSs although the AUC of EU-TIRADS showed the numerically lowest value.

## 4. Discussion

We selected 1143 ND/UNS samples from 16,321 thyroid aspiration samples collected over a five-year period at a tertiary referral center. In these ND/UNS nodules, we estimated the malignancy/benign rates according to the categories of different US-based RSSs and compared the diagnostic performance of US-based RSSs. The ATA nodule sonographic pattern system, K-TIRADS, ACR TIRADS, and EU-TIRADS were effective in differentiating cases with a high ROM and reliably predicting thyroid malignancy among ND/UNS nodules, suggesting their role as useful diagnostic tools in these diagnostically challenging nodules. However, considering parameters including LRs, the EU-TIRADS demonstrated inferior diagnostic performance in predicting malignancy for ND/UNS nodules. In real-world practice, for ND/UNS nodules, cytological or histopathological follow-up was more common in the higher categories of US-based RSSs.

In this study, 7.79% of ND/UNS nodules were surgically resected, and the minimum malignancy rate of ND/UNS nodules was 3.41%, slightly above the upper limit of the ROM for benign cytology (0–3%). This level is lower than the suggested overall ROM for ND/UNS specimens in the 2017 TBSRTC (5–10%) [16] but within the 1–4% risk range recommended in the previous version of TBSRTC [15]. Considering that clinically suspicious nodules are more likely to undergo surgical resection early, it is reasonable to calculate the malignancy rate based on the total number of FNA cases [28]. Although previous studies reported a higher ROM (at least 5.3%) for ND/UNS nodules [17,24], they included cases with available follow-up CNB results only [24] or selected surgically-excised nodules, nodules with diagnostic results at repeat FNA, cases with nondiagnostic results at repeat FNA but no increase in size, or cases with a stable or decreased size during follow-up only [17]. Therefore, the possibility of ROM overestimation due to selection bias should be considered since nodules without histopathological or cytological follow-up, particularly those without even radiological follow-up, are more likely to be cases with low clinical suspicion. Therefore, it might be reasonable to estimate the overall ROM for ND/UNS nodules encountered in routine clinical practice as <5%.

In our study, cytological or histopathological follow-ups for ND/UNS nodules were more common in cases with higher categories of US-based RSSs. This suggests that although US-based RSSs were helpful in predicting benign diagnosis, further evaluations to confirm a benign or malignant status were increased in advanced categories of US-based RSSs. Because of this phenomenon, the benign rates defined through the cytological or histopathological follow-up results did not concordantly match the categories of US-based RSSs. The proportion of benign cases proven through cytological or histopathological follow-up was only 10.06% (minimum benign rate). However, only 18.02% of the ND/UNS nodules in our study were followed-up cytologically or histopathologically. In the remaining 81.98%, repeat FNA, CNB, and/or surgical resection were not applied, and most of them are considered to be managed conservatively due to a lack of worrisome clinical/sonographic features. Although TBSRTC guidelines recommend repeat FNA with US guidance for ND/UNS nodules [16], in real practice, most ND/UNS nodules, especially those with low categories of US-based RSSs, were observed without repeat FNA, as nodules with benign cytology. This is the reality of clinical practice that has not been reported before. Making decisions on nodules with uncertain cytological data refers to “unknown knowns” (things we understand but are not aware of) [29,30]. When dealing with “unknown knowns”, despite uncertainty, to efficiently utilize available resources and time, strict diagnostic efforts may be reduced for a significant proportion of ND/UNS nodules based on low category information of US-based RSSs. This is consistent with the current trend in clinical practice for thyroid nodules in which diagnostic and therapeutic interventions are focused on high-risk groups and conservative approaches are applied for low-risk groups.

The maximum benign rate of category 5 was less than half of that in category 4 of the ATA nodule sonographic pattern system, K-TIRADS, and ACR TIRADS. This phenomenon was not demonstrated when the EU-TIRADS was applied for ND/UNS nodules. This suggests the excellence of the highest category of the ATA nodule sonographic pattern system, K-TIRADS, and ACR TIRADS in predicting thyroid malignancy from ND/UNS nodules.

Category 5 in the ATA nodule sonographic pattern system, K-TIRADS, and ACR TIRADS demonstrated a positive LR of >5. Stated in another way, malignant nodules were >5 times more likely to be in category 5 of these three US-based RSSs than were nonmalignant nodules. Therefore, without priority, these three systems may be useful diagnostic tests to guide the further management of ND/UNS nodules. However, category 5 in the EU-TIRADS exhibited a positive LR of only 2.214, demonstrating inferior performance compared with the other systems. The criteria to be classified as category 5 are the least demanding for the EU-TIRADS compared to the other systems. It is sufficient for a nodule to be classified as category 5 of EU-TIRADS if it has one of the following four features: irregular shape (nonparallel orientation), irregular (spiculated/microlobulated) margin, microcalcifications, and marked hypoechogenicity (and solid) [5]. However, in K-TIRADS [2], to be classified as category 5, a nodule needs to be solid hypoechoic and ≥one of the three suspicious features (microcalcifications, nonparallel orientation, spiculated/microlobulated margin) should be present. Likewise, according to the ATA guidelines [4], to be stratified as a high suspicion nodule, it must be a solid hypoechoic nodule or solid hypoechoic component of a partially cystic nodule and at the same time, ≥one of the following features should be accompanied: irregular margin, microcalcification, nonparallel orientation, rim calcification with small extrusive soft tissue component, and evidence of extrathyroidal extension. Therefore, marked hypoechoic solid nodules without other suspicious features (category 4 in ATA nodule sonographic pattern system and K-TIRADS) and isoechoic/hyperechoic nodules with microcalcifications, nonparallel orientation, or spiculated/microlobulated margin (unclassifiable in ATA nodule sonographic pattern system and category 4 in K-TIRADS) are classified into category 5 only in EU-TIRADS. The ACR TIRADS has a totally different system, which allocates points for every US feature of a nodule, and the nodule ACR TIRADS level is defined based on the total sum of the points [3]. Although the presence of marked hypoechogenicity, nonparallel orientation, irregular margin, or punctate echogenic foci suggesting the possibility of microcalcification increases the points, presence of only one feature does not produce sufficient points to reach the ACR TIRADS 5. With respect to EU-TIRADS, these least stringent criteria to be stratified as category 5 may be associated with the highest number of cases in category 5 (Table S2) and the lowest positive LR of 2.214 in diagnosing malignancy for category ≥ 5, compared to the other three RSSs. Furthermore, differences in the epidemiology of thyroid tumors between Korea and Europe may have also partly affected the results. Each US-based RSS was established for its local population and the epidemiology of thyroid tumors may be varied by the iodine intake and/or genetic variations [31]. In Korea, where our study was conducted, iodine intake is more than adequate, and PTCs were reported to constitute 92% of thyroid cancers, while FTCs account for only approximately 3% [13,32]. In the current study, although the proportion of FTCs among the excised ND/UNS nodules turned out to be malignant (15.4%) was higher than the proportion among the total thyroid cancers reported in Korea, this proportion of FTCs is still much lower than that reported in European countries (27–37%) [33,34,35,36]. In Korea, application of the EU-TIRADS may be less appropriate, as follicular proliferative lesions, including follicular adenomas, FTCs, and NIFTPs, are much rarer than in Europe. Recently, the International Thyroid Nodule Ultrasound Working Group, standing for major thyroid associations, including the ATA, is developing the universal guidelines on the US-based stratification of thyroid nodules, called the U-TIRADS [37]. In developing this U-TIRADS, regional epidemiological variations in thyroid tumor histology may need to be contemplated.

Several limitations of this study should be acknowledged. First, analyses were performed per nodule, not per patient, which may have led to an overestimation of our results. However, when the same analyses were repeated after excluding cases where ≥two nodules were included from one patient, the findings were not changed. Second, we analyzed the positive and negative LRs and ROC curves of US-based RSSs for predicting thyroid malignancy only in surgically-resected ND/UNS nodules since the gold standard diagnosis based on surgical pathology is required to evaluate the performance of diagnostic tests. Therefore, this information is applicable to cases with high clinical suspicion rendered to surgery, not all ND/UNS nodules, most of which are managed conservatively. Third, our study is based on samples from Korea, an iodine-sufficient area. Therefore, caution should be applied before extrapolating our findings to countries with different dietary iodine contents and/or thyroid tumor epidemiology. Fourth, US-based RSSs that include new US parameters such as elastography or 4-dimensional (4D) vascularity [31] were not evaluated in the current study.

## 5. Conclusions

In conclusion, this real-world comparative study demonstrated the following important findings on the diagnostic performance and clinical applications of US-based RSSs for thyroid nodules with ND/UNS cytology. For ND/UNS nodules, although US-based RSSs aided in predicting benign lesions, in real-world practice, further diagnostic evaluations to confirm the benign status, including repeat FNA, were increased in higher categories of US-based RSSs. The ATA nodule sonographic pattern system, K-TIRADS, and ACR TIRADS, especially the highest category of these systems, were more competent in predicting malignancy from ND/UNS nodules. These systems may be useful diagnostic tools to guide the further management of ND/UNS nodules. The EU-TIRADS, although it was also helpful, exhibited relatively less effective diagnostic performance in predicting malignancy for nodules with ND/UNS cytology in Korea, where iodine intake is more than adequate. These findings have implications for developing and verifying the U-TIRADS and applying the U-TIRADS to ND/UNS nodules.

## Figures and Tables

**Table 1:** Baseline characteristics of the study population and included thyroid nodules with nondiagnostic or unsatisfactory cytopathological results.

Variable	Total Enrollment	Cases of Surgical Resection
Patient characteristics		
Number	1125	87
Age (years)		
Median (interquartile range)	54.0 (45.0–61.0)	52.0 (42.3–59.0)
Range	14.0–88.0	22.0–82.0
Female [n (%)]	843 (74.90)	64 (73.56)
Nodule characteristics		
Number	1143	89
Nodule size (cm)		
Median (interquartile range)	1.30 (0.83–2.30)	1.80 (0.80–3.90)
Range	0.30–12.00	0.30–12.00
Surgically resected [n (%)]	89 (7.79)	89 (100.00)

Continuous variables with nonnormal distributions are expressed as the median (interquartile range). Cases were collected from April 2011 to March 2016.

**Table 2:** Malignancy rates of thyroid nodules with nondiagnostic or unsatisfactory cytopathological results according to categories of ultrasonography-based risk stratification systems.

US-Based Risk Stratification Systems	All FNAs (Number)	Surgically Resected Nodules (Number)	Malignant Nodules Confirmed by Surgery (Number)	% Malignant	
				% Maximum *	% Minimum ^†^
EU-TIRADS					
2 (benign)	11	0	0	N/A	0.00
3 (low risk)	589	39	11	28.21	1.87
4 (intermediate risk)	300	20	9	45.00	3.00
5 (high risk)	243	30	19	63.33	7.82
ATA nodule sonographic pattern system					
Not applicable ^‡^	87	3	1	33.33	1.15
1 (benign)	9	0	0	N/A	0.00
2 (very low suspicion)	65	2	1	50.00	1.54
3 (low suspicion)	547	38	10	26.32	1.83
4 (intermediate suspicion)	329	25	10	40.00	3.04
5 (high suspicion)	106	21	17	80.95	16.03
ACR TIRADS					
1 (benign)	101	5	2	40.00	1.98
2 (not suspicious)	238	20	3	15.00	1.26
3 (mildly suspicious)	240	11	5	45.45	2.08
4 (moderate suspicious)	452	33	13	39.39	2.88
5 (highly suspicious)	112	20	16	80.00	14.29
K-TIRADS					
2 (benign)	71	2	1	50.00	1.41
3 (low suspicion)	550	38	10	26.32	1.82
4 (intermediate suspicion)	417	28	11	39.29	2.64
5 (high suspicion)	105	21	17	80.95	16.19
Total	1143	89	39	43.82	3.41

US = ultrasonography, FNA = fine-needle aspiration, TIRADS = Thyroid Imaging, Reporting and Data System, ATA = American Thyroid Association, ACR = American College of Radiology. * Percentage of cases calculated from the total number of resected cases in each category (maximum malignancy rate). ^†^ Percentage of cases calculated from the total number of fine-needle aspirations in each category (minimum malignancy rate). ^‡^ Isoechoic/hyperechoic nodules with one or more of the following features: irregular margins, microcalcification, taller-than-wide shape, rim calcification with small extrusive soft tissue component, and evidence of extrathyroidal extension.

**Table 3:** Benign rates of thyroid nodules with nondiagnostic or unsatisfactory cytopathological results according to categories of ultrasonography-based risk stratification systems.

US-Based Risk Stratification Systems	All FNAs (Number)	Nodules with Cytological or Histopathological Follow-up (Number)	Nodules with Final Diagnosis of Benign (Number)	% Benign	
				% Maximum *	% Minimum ^†^
EU-TIRADS					
2 (benign)	11	0	0	N/A	0.00
3 (low risk)	589	88	49	55.68	8.32
4 (intermediate risk)	300	56	37	66.07	12.33
5 (high risk)	243	62	29	46.77	11.93
ATA nodule sonographic pattern system					
Not applicable ^‡^	87	13	8	61.54	9.20
1 (benign)	9	0	0	N/A	0.00
2 (very low suspicion)	65	6	3	50.00	4.62
3 (low suspicion)	547	84	47	55.95	8.59
4 (intermediate suspicion)	329	66	45	68.18	13.68
5 (high suspicion)	106	37	12	32.43	11.32
ACR TIRADS					
1 (benign)	101	11	6	54.55	5.94
2 (not suspicious)	238	43	24	55.81	10.08
3 (mildly suspicious)	240	25	12	48.00	5.00
4 (moderate suspicious)	452	91	61	67.03	13.50
5 (highly suspicious)	112	36	12	33.33	10.71
K-TIRADS					
2 (benign)	71	8	3	37.50	4.23
3 (low suspicion)	550	82	47	57.32	8.55
4 (intermediate suspicion)	417	80	54	67.50	12.95
5 (high suspicion)	105	36	11	30.56	10.48
Total	1143	206	115	55.83	10.06

US = ultrasonography, FNA = fine-needle aspiration, TIRADS = Thyroid Imaging, Reporting and Data System, ATA = American Thyroid Association, ACR = American College of Radiology. * Percentage of cases calculated from the total number of nodules with cytological or histopathological follow-up in each category (maximum rate). ^†^ Percentage of cases calculated from the total number of fine-needle aspirations in each category (minimum rate). ^‡^ Isoechoic/hyperechoic nodules with one or more of the following features: irregular margins, microcalcification, taller-than-wide shape, rim calcification with small extrusive soft tissue component, and evidence of extrathyroidal extension.

**Table 4:** Likelihood ratios for various ultrasonography-based risk stratification systems in diagnosing thyroid malignancy in nodules with nondiagnostic or unsatisfactory cytopathological results.

Cutoff	Malignant Nodules Confirmed by Surgery (Number)	Nonmalignant Nodules Confirmed by Surgery (Number)	Positive LR (95% CI)	Negative LR (95% CI)
EU-TIRADS				
≥3	39	50	1.000 (0.953, 1.043)	0
≥4	28	22	**1.632 (1.128, 2.361)**	**0.504 (0.288, 0.880)**
≥5	19	11	**2.214 (1.199, 4.089)**	**0.657 (0.468, 0.923)**
Total	39	50		
ATA nodule sonographic pattern system *				
≥2	38	48	1.000 (0.952, 1.045)	0
≥3	37	47	0.994 (0.930, 1.063)	1.263 (0.082, 19.543)
≥4	27	19	**1.795 (1.198, 2.689)**	**0.479 (0.277, 0.829)**
≥5	17	4	**5.368 (1.970, 14.631)**	**0.603 (0.447, 0.813)**
Total	38	48		
ACR TIRADS				
≥1	39	50	1.000 (0.953–1.043)	0
≥2	37	47	1.009 (0.912–1.117)	0.855 (0.150, 4.867)
≥3	34	30	**1.453 (1.124, 1.878)**	**0.321 (0.132, 0.777)**
≥4	29	24	**1.549 (1.100, 2.182)**	**0.493 (0.271, 0.896)**
≥5	16	4	**5.128 (1.863, 14.115)**	**0.641 (0.487, 0.843)**
Total	39	50		
K-TIRADS				
≥2	39	50	1.000 (0.953, 1.043)	0
≥3	38	49	0.994 (0.932, 1.060)	1.282 (0.083, 19.856)
≥4	28	21	**1.709 (1.168, 2.501)**	**0.486 (0.280, 0.846)**
≥5	17	4	**5.449 (1.993, 14.893)**	**0.613 (0.460, 0.818)**
Total	39	50		

LR = likelihood ratio, TIRADS = Thyroid Imaging, Reporting and Data System, ATA = American Thyroid Association, ACR = American College of Radiology. * Nonapplicable cases (isoechoic nodule with one or more of the following features: irregular margins, microcalcification, taller-than-wide shape, rim calcification with small extrusive soft tissue component, and evidence of extrathyroidal extension) were excluded from analysis. Statistically significant values were boldfaced.

**Table 5:** Area under the receiver operating characteristic curve of ultrasonography-based risk stratification systems to predict thyroid malignancy in nodules with nondiagnostic or unsatisfactory cytopathological results.

US-Based Risk Stratification Systems	Area under ROC Curve (95% CI)	*p*
EU-TIRADS	0.667 (0.559, 0.764)	**0.0022**
ATA nodule sonographic pattern system *	0.711 (0.604, 0.804)	**0.0001**
ACR TIRADS	0.712 (0.606, 0.803)	**0.0001**
K-TIRADS	0.707 (0.601, 0.799)	**0.0001**

US = ultrasound, ROC = receiver operating characteristic, CI = confidence interval, TIRADS = Thyroid Imaging, Reporting and Data System, ATA = American Thyroid Association, ACR = American College of Radiology. * Nonapplicable cases (isoechoic/hyperechoic nodule with one or more of the following features: irregular margins, microcalcification, taller-than-wide shape, rim calcification with small extrusive soft tissue component, evidence of extrathyroidal extension) were excluded from analysis. Statistically significant values were boldfaced.

**Table 6:** Pairwise comparison of the area under the receiver operating characteristic curve of ultrasonography-based risk stratification systems to predict thyroid malignancy in nodules with nondiagnostic or unsatisfactory cytopathological results.

	Difference in Area (95% CI)	*p*
ACR TIRADS−EU-TIRADS	0.045 (−0.012, 0.102)	0.1213
ACR TIRADS−K-TIRADS	0.005 (−0.040, 0.050)	0.8323
ACR TIRADS−ATA nodule sonographic pattern system *	0.006 (−0.041, 0.053)	0.8015
K-TIRADS−EU-TIRADS	0.040 (−0.002, 0.082)	0.0600
K-TIRADS−ATA nodule sonographic pattern system *	0.000 (0.000, 0.000)	1.0000
ATA nodule sonographic pattern system−EU-TIRADS *	0.032 (−0.006, 0.071)	0.0970

ROC = receiver operating characteristic, CI = confidence interval. * Nodules not applicable to the ATA sonographic pattern system (isoechoic/hyperechoic nodule with one or more of the following features: irregular margins, microcalcification, taller-than-wide shape, rim calcification with small extrusive soft tissue component, and evidence of extrathyroidal extension) were excluded from analysis.

## Data Availability

Restrictions apply to the availability of these data. Data was obtained from Samsung Medical Center and are available from the corresponding authors with the permission of Samsung Medical Center.

## Supplementary Materials

The following is available online at https://www.mdpi.com/article/10.3390/cancers13081948/s1, Table S1: Histologic subtypes of excised nodules with nondiagnostic/unsatisfactory cytology; Table S2: Frequencies of ultrasound imaging characteristics and EU-TIRADS categories in included thyroid nodules with nondiagnostic/unsatisfactory cytology.

## Abbreviations

ACR	American College of Radiology
ATA	American Thyroid Association
AUC	area under the curve
CNB	core needle biopsy
ETA	European Thyroid Association
EU	European
FNA	fine-needle aspiration
IRB	Institutional Review Board
K	Korean
KTA/KSThR	Korean Thyroid Association/Korean Society of Thyroid Radiology
LR	likelihood ratio
NIFTP	Noninvasive follicular thyroid neoplasms with papillary-like nuclear feature
ND/UNS	nondiagnostic/unsatisfactory
ROC	receiver operating characteristic
ROM	risk of malignancy
RSS	risk stratification system
TBSRTC	the Bethesda system for reporting thyroid cytopathology
TIRADS	Thyroid Imaging, Reporting and Data System
US	ultrasound

## References

[1] Ha E.J., Baek J.H., Na D.G. (2017). Risk Stratification of Thyroid Nodules on Ultrasonography: Current Status and Perspectives. Thyroid.

[2] Shin J.H., Baek J.H., Chung J., Ha E.J., Kim J.H., Lee Y.H., Lim H.K., Moon W.J., Na D.G., Park J.S. (2016). Ultrasonography Diagnosis and Imaging-Based Management of Thyroid Nodules: Revised Korean Society of Thyroid Radiology Consensus Statement and Recommendations. Korean J. Radiol..

[3] Tessler F.N., Middleton W.D., Grant E.G., Hoang J.K., Berland L.L., Teefey S.A., Cronan J.J., Beland M.D., Desser T.S., Frates M.C. (2017). ACR Thyroid Imaging, Reporting and Data System (TI-RADS): White Paper of the ACR TI-RADS Committee. J. Am. Coll. Radiol..

[4] Haugen B.R., Alexander E.K., Bible K.C., Doherty G.M., Mandel S.J., Nikiforov Y.E., Pacini F., Randolph G.W., Sawka A.M., Schlumberger M. (2016). 2015 American Thyroid Association Management Guidelines for Adult Patients with Thyroid Nodules and Differentiated Thyroid Cancer: The American Thyroid Association Guidelines Task Force on Thyroid Nodules and Differentiated Thyroid Cancer. Thyroid.

[5] Russ G., Bonnema S.J., Erdogan M.F., Durante C., Ngu R., Leenhardt L. (2017). European Thyroid Association Guidelines for Ultrasound Malignancy Risk Stratification of Thyroid Nodules in Adults: The EU-TIRADS. Eur. Thyroid J..

[6] Ha E.J., Na D.G., Baek J.H., Sung J.Y., Kim J.H., Kang S.Y. (2018). US Fine-Needle Aspiration Biopsy for Thyroid Malignancy: Diagnostic Performance of Seven Society Guidelines Applied to 2000 Thyroid Nodules. Radiology.

[7] Ha E.J., Na D.G., Moon W.J., Lee Y.H., Choi N. (2018). Diagnostic Performance of Ultrasound-Based Risk-Stratification Systems for Thyroid Nodules: Comparison of the 2015 American Thyroid Association Guidelines with the 2016 Korean Thyroid Association/Korean Society of Thyroid Radiology and 2017 American College of Radiology Guidelines. Thyroid.

[8] Castellana M., Castellana C., Treglia G., Giorgino F., Giovanella L., Russ G., Trimboli P. (2020). Performance of five ultrasound risk stratification systems in selecting thyroid nodules for FNA. J. Clin. Endocrinol. Metab..

[9] Lauria Pantano A., Maddaloni E., Briganti S.I., Beretta Anguissola G., Perrella E., Taffon C., Palermo A., Pozzilli P., Manfrini S., Crescenzi A. (2018). Differences between ATA, AACE/ACE/AME and ACR TI-RADS ultrasound classifications performance in identifying cytological high-risk thyroid nodules. Eur. J. Endocrinol..

[10] Middleton W.D., Teefey S.A., Reading C.C., Langer J.E., Beland M.D., Szabunio M.M., Desser T.S. (2018). Comparison of Performance Characteristics of American College of Radiology TI-RADS, Korean Society of Thyroid Radiology TIRADS, and American Thyroid Association Guidelines. AJR Am. J. Roentgenol..

[11] Grani G., Lamartina L., Ascoli V., Bosco D., Biffoni M., Giacomelli L., Maranghi M., Falcone R., Ramundo V., Cantisani V. (2019). Reducing the Number of Unnecessary Thyroid Biopsies While Improving Diagnostic Accuracy: Toward the “Right” TIRADS. J. Clin. Endocrinol. Metab..

[12] Yoon S.J., Na D.G., Gwon H.Y., Paik W., Kim W.J., Song J.S., Shim M.S. (2019). Similarities and Differences Between Thyroid Imaging Reporting and Data Systems. AJR Am. J. Roentgenol..

[13] Lee Y.B., Cho Y.Y., Jang J.Y., Kim T.H., Jang H.W., Chung J.H., Oh Y.L., Kim S.W. (2017). Current status and diagnostic values of the Bethesda system for reporting thyroid cytopathology in a papillary thyroid carcinoma-prevalent area. Head Neck.

[14] Bongiovanni M., Spitale A., Faquin W.C., Mazzucchelli L., Baloch Z.W. (2012). The Bethesda System for Reporting Thyroid Cytopathology: A meta-analysis. Acta Cytol..

[15] Cibas E.S., Ali S.Z. (2009). The Bethesda System For Reporting Thyroid Cytopathology. Am. J. Clin. Pathol..

[16] Cibas E.S., Ali S.Z. (2017). The 2017 Bethesda System for Reporting Thyroid Cytopathology. Thyroid.

[17] Moon H.J., Kim E.K., Yoon J.H., Kwak J.Y. (2015). Malignancy risk stratification in thyroid nodules with nondiagnostic results at cytologic examination: Combination of thyroid imaging reporting and data system and the Bethesda System. Radiology.

[18] Lee Y.B., Kim J.Y., Cho H., Hahn S.Y., Shin J.H., Lee S.E., Jun J.E., Kim S.W., Chung J.H., Kim T.H. (2018). Modified Bethesda system informing cytopathologic adequacy improves malignancy risk stratification in nodules considered benign or atypia (follicular lesion) of undetermined significance. Sci. Rep..

[19] Renshaw A.A. (2011). Non-diagnostic rates for thyroid fine needle aspiration are negatively correlated with positive for malignancy rates. Acta Cytol..

[20] Hong M.J., Na D.G., Baek J.H., Sung J.Y., Kim J.H. (2017). Cytology-Ultrasonography Risk-Stratification Scoring System Based on Fine-Needle Aspiration Cytology and the Korean-Thyroid Imaging Reporting and Data System. Thyroid.

[21] Park C.J., Kim E.K., Moon H.J., Yoon J.H., Park V.Y., Kwak J.Y. (2018). Thyroid Nodules With Nondiagnostic Cytologic Results: Follow-Up Management Using Ultrasound Patterns Based on the 2015 American Thyroid Association Guidelines. AJR Am. J. Roentgenol..

[22] Ho A.S., Sarti E.E., Jain K.S., Wang H., Nixon I.J., Shaha A.R., Shah J.P., Kraus D.H., Ghossein R., Fish S.A. (2014). Malignancy rate in thyroid nodules classified as Bethesda category III (AUS/FLUS). Thyroid.

[23] Nikiforov Y.E., Seethala R.R., Tallini G., Baloch Z.W., Basolo F., Thompson L.D., Barletta J.A., Wenig B.M., Al Ghuzlan A., Kakudo K. (2016). Nomenclature Revision for Encapsulated Follicular Variant of Papillary Thyroid Carcinoma: A Paradigm Shift to Reduce Overtreatment of Indolent Tumors. JAMA Oncol..

[24] Yeon J.S., Baek J.H., Lim H.K., Ha E.J., Kim J.K., Song D.E., Kim T.Y., Lee J.H. (2013). Thyroid nodules with initially nondiagnostic cytologic results: The role of core-needle biopsy. Radiology.

[25] Grimes D.A., Schulz K.F. (2005). Refining clinical diagnosis with likelihood ratios. Lancet.

[26] Altman D.G., Bland J.M. (1994). Diagnostic tests 2: Predictive values. BMJ.

[27] DeLong E.R., DeLong D.M., Clarke-Pearson D.L. (1988). Comparing the areas under two or more correlated receiver operating characteristic curves: A nonparametric approach. Biometrics.

[28] Kim T.H., Jeong D.J., Hahn S.Y., Shin J.H., Oh Y.L., Ki C.S., Kim J.W., Jang J.Y., Cho Y.Y., Chung J.H. (2016). Triage of patients with AUS/FLUS on thyroid cytopathology: Effectiveness of the multimodal diagnostic techniques. Cancer Med.

[29] Girard J.P., Girard J.L., Girard J.P., Girard J.L. (2009). Simple ideas that work in complex environments. A Leader’s Guide to Knowledge Management: Drawing on the Past to Enhance Future Performance.

[30] Rumsfeld D. United States Secretary of Defense.

[31] Borlea A., Borcan F., Sporea I., Dehelean C.A., Negrea R., Cotoi L., Stoian D. (2020). TI-RADS Diagnostic Performance: Which Algorithm is Superior and How Elastography and 4D Vascularity Improve the Malignancy Risk Assessment. Diagnostics.

[32] Cho Y.Y., Lim J., Oh C.M., Ryu J., Jung K.W., Chung J.H., Won Y.J., Kim S.W. (2015). Elevated risks of subsequent primary malignancies in patients with thyroid cancer: A nationwide, population-based study in Korea. Cancer.

[33] Hölzer S., Reiners C., Mann K., Bamberg M., Rothmund M., Dudeck J., Stewart A.K., Hundahl S.A. (2000). Patterns of care for patients with primary differentiated carcinoma of the thyroid gland treated in Germany during 1996. U.S. and German Thyroid Cancer Group. Cancer.

[34] Machens A., Holzhausen H.J., Dralle H. (2005). The prognostic value of primary tumor size in papillary and follicular thyroid carcinoma. Cancer.

[35] Passler C., Scheuba C., Prager G., Kaczirek K., Kaserer K., Zettinig G., Niederle B. (2004). Prognostic factors of papillary and follicular thyroid cancer: Differences in an iodine-replete endemic goiter region. Endocr. Relat. Cancer.

[36] Santos J.E., Freitas M., Fonseca C.P., Castilho P., Carreira I.M., Rombeau J.L., Branco M.C. (2017). Iodine deficiency a persisting problem: Assessment of iodine nutrition and evaluation of thyroid nodular pathology in Portugal. J. Endocrinol. Invest..

[37] Luster M., Aktolun C., Amendoeira I., Barczyński M., Bible K.C., Duntas L.H., Elisei R., Handkiewicz-Junak D., Hoffmann M., Jarząb B. (2019). European Perspective on 2015 American Thyroid Association Management Guidelines for Adult Patients with Thyroid Nodules and Differentiated Thyroid Cancer: Proceedings of an Interactive International Symposium. Thyroid.

