# Supplementary material for: Comparison of Four Ultrasonography-Based Risk Stratification Systems in Thyroid Nodules with Nondiagnostic/Unsatisfactory Cytology: A Real-World Study
Source: Cancers (Basel). 2021 Apr 18;13(8):1948. doi: 10.3390/cancers13081948 (PMC8073392; doi:10.3390/cancers13081948)
Supplement: Supplementary file 1 [file cancers-13-01948-s001.zip › cancers-1160302-SI.pdf]

cancers

MDPI

Article

Comparison of Four Ultrasonography-Based Risk Stratification Systems in Thyroid Nodules with Nondiagnostic/Unsatisfactory Cytology: A Real-World Study

You-Bin Lee, Young Lyun Oh, Jung Hee Shin, Sun Wook Kim, Jae Hoon Chung, Yong-Ki Min, Soo Yeon Hahn and Tae Hyuk Kim

Table S1. Histologic subtypes of excised nodules with nondiagnostic/unsatisfactory cytology.

Histologic Subtype	Number of Nodules (%)
Malignant	
Total	39 (100.0)
Papillary thyroid carcinoma	32 (82.1)
Follicular thyroid carcinoma	6 (15.4)
Medullary thyroid carcinoma	1 (2.6)
Nonmalignant	
Total	50 (100.0)
Benign follicular nodule	30 (60.0)
Follicular adenoma	20 (40.0)

Table S2. Frequencies of ultrasound imaging characteristics and EU-TIRADS categories in included thyroid nodules with nondiagnostic/unsatisfactory cytology

Ultrasound imaging characteristics	Number of nodules (%)
Composition	
Solid	688 (60.2)
Predominantly solid	316 (27.6)
Predominantly cystic	129 (11.3)
Cystic	10 (0.9)
Echogenicity	
Hyperechoic	2 (0.2)
Isoechoic	677 (59.2)
Mild hypoechoic	392 (34.3)
Marked hypoechoic	62 (5.4)
N/A (pure cyst)	10 (0.9)
Orientation	
Parallel	1,075 (94.1)
Non-parallel	68 (5.9)
Margin	
Smooth	548 (47.9)
Ill-defined	554 (48.5)
spiculated/microlobulated	41 (3.6)
Calcification	
Microcalcification	129 (11.3)
Other calcifications (macrocalcification or rim calcification) without microcalcification	223 (19.5)

No calcification	791 (69.2)
Final EU-TIRADS category	
2 (benign)	11 (1.0)
3 (low risk)	589 (51.5)
4 (intermediate risk)	300 (26.2)
5 (high risk)	243 (21.3)
Total	1,143 (100.0%)

EU= European, TIRADS = Thyroid Imaging, Reporting and Data System.
